# Dietary Practices during Pregnancy in a Marshallese Community: A Mixed Methods Analysis

**DOI:** 10.3390/ijerph19116360

**Published:** 2022-05-24

**Authors:** Britni L. Ayers, Cari A. Bogulski, Ashlea Bennett-Milburn, Anna Fisher, Morda Netwon, Pearl A. McElfish

**Affiliations:** 1 College of Medicine, University of Arkansas for Medical Sciences Northwest, 1125 N. College Avenue, Fayetteville, AR 72703, USA; cbogulski@uams.edu (C.A.B.); mnetwon@uams.edu (M.N.); pamcelfish@uams.edu (P.A.M.); 2 Bell Engineering Center, University of Arkansas Fayetteville, 800 W. Dickson St., Fayetteville, AR 72701, USA; ashlea@uark.edu; 3 Department of Pediatrics, The University of Utah, 295 Chipeta Way, Salt Lake City, UT 84103, USA; anna.fisher@hsc.utah.edu

**Keywords:** dietary practices, infant health, Marshallese, maternal health, pregnancy

## Abstract

Dietary practices during pregnancy play a pivotal role in the health of women and their children and set the foundation for long-term health. Marshallese women have disproportionally higher rates of maternal and infant health disparities, yet little is known about the dietary practices during their pregnancy. The purpose of this study was to identify dietary practices during pregnancy among Marshallese women. From March 2019 to March 2020, a purposive sample of 33 pregnant Marshallese participants participated in a mixed methods study. Two primary themes emerged: (1) traditional beliefs about a healthy diet during pregnancy; and (2) dietary change during pregnancy. Within the first theme, four subthemes emerged: (1) should eat; (2) should not eat; (3) challenges to traditional diet; and (4) spiritual dietary customs during pregnancy. Within the second theme, three subthemes emerged: (1) a healthy diet for my baby; (2) autonomy and diet; and (3) sugar-sweetened beverages. The transition in discourse from traditional customs of dietary practices to an individualistic discourse highlights that acculturation is a complex process that should be included in maternal health education and interventions. Findings from this study provide insight into potential considerations for future interventions aiming to improve maternal and child health outcomes among Marshallese.

## 1. Introduction

Dietary practices during pregnancy play a pivotal role in the health and wellbeing of women and their children and set the foundation for long-term health for both the mother and child [1]. The World Health Organization (WHO) has established dietary guidelines and recommendations for nutritional intake to better ensure a healthy pregnancy and reduce maternal and infant health disparities [2].

Prenatal dietary practices can be shaped by cultural values, religion, sociodemographic factors, and migration [3,4]. Studies on migrant populations have shown that migration from one country to another is associated with changes in dietary practices and an adoption of the cultural norms of the host country, a phenomenon referred to as acculturation [5,6].

Pacific Islanders are the second-fastest growing migrant population in the United States (US). The majority of growth in the Pacific Islander migrant population (66%) occurs in the south, especially in Arkansas, where the annual growth rate is 252% and the majority of Pacific Islanders are Marshallese [7]. Pacific Islander women residing in the US, and Marshallese women specifically, have disproportionally higher rates of preterm birth (<37 completed weeks) and lower birthweight infants (<2500 g) and are also more likely to experience preeclampsia, primary cesarean delivery, excessive gestational weight gain, gestational diabetes mellitus (GDM), and low exclusive breastfeeding initiation and duration at six months compared to other racial/ethnic minorities and the US population in general [8,9,10,11,12,13,14,15].

Despite the increase in migration and the adverse maternal and infant health outcomes among Marshallese or other Pacific Islanders, little is known about their dietary practices during pregnancy or how their dietary practices have changed after moving to the US. Understanding changes in dietary practices as a result of migration may inform the cultural tailoring of educational programs and interventions to improve maternal and child health outcomes. There are currently no nutrition interventions that target Marshallese pregnant women in the US, identifying a gap in service for this community.

The authors used a community-based participatory research (CBPR) approach to address the health disparities among the Marshallese residing in Arkansas. CBPR is a research approach seeking to involve community partners in all aspects of the research process [16]. This type of research is uniquely suited for engaging indigenous and/or immigrant populations. As part of the CBPR collaborative, the research team has spent the past six years meeting with Marshallese community members to determine and prioritize the community’s primary health concerns [17,18,19,20,21]. Maternal health was identified as a top priority. The purpose of this study is to identify dietary practices during pregnancy in a Marshallese community residing in Arkansas.

## 2. Methods

### 2.1. Recruitment and Sampling

All study documents used for recruitment, consent, and retention were developed in collaboration with Marshallese stakeholders using a CBPR approach. Stakeholders included a community action network, which was comprised of Marshallese community members and health care services providers, and community health workers (CHWs). Participants were recruited by female bilingual CHWs with extensive research training and trust within the Marshallese community. Thirty-three women were recruited by the CHWs at local clinics, faith-based organizations, and community-based organizations. The target recruitment was 30 participants. From prior work, 30 participants were sufficient to achieve saturation across a diverse group of Marshallese women in Arkansas [1,6]. Saturation occurs when redundancy is reached in data analysis and signals to researchers that data collection may cease [22]. The inclusion criteria were: (1) women who self-report as Marshallese; (2) 18 years of age or older; and (3) pregnant. Exclusion criteria were: (1) conception with the use of fertility treatments; (2) multiple gestations; and (3) use of medications known to influence fetal growth (e.g., glucocorticoids, insulin, thyroid, hormones). These exclusion criteria were chosen because these components would qualify the participants for potential high-risk pregnancies. All study procedures and materials were approved by the University of Arkansas for Medical Sciences Institutional Review Board (#228023).

Potential participants who met the inclusion criteria were offered the opportunity to join the study and completed the consent process. Trained CHWs provided each participant a copy of the consent in either/both English and/or Marshallese. The consent forms used plain language. The CHWs read the consent aloud to the participants in the participants’ language of choice (English or Marshallese). Participants were given the opportunity to ask questions and have their questions answered prior to consent from either the CHWs or the Principal Investigator of the study. Participants were also provided the contact information of the Principal Investigator if any questions arose throughout the study. Participants received a $40 gift card at the data collection event.

### 2.2. Research Design

A mixed methods design was chosen to ensure a more comprehensive view of participants’ dietary practices during pregnancy and to overcome the limitations of a single design [23]. The mixed methods design consisted of a survey for quantitative analysis and individual participant interviews for qualitative analysis (see “Supplementary Materials”) [24]. Survey and individual interview guides were developed in collaboration with our Marshallese Community Advisory Board and validated for this particular population. Both instruments were piloted with Marshallese staff. The survey went through four iterations, and the interview guide went through three iterations.

The purpose of the quantitative portion of the study was to characterize participants’ dietary practices during pregnancy. The surveys were implemented using Research Electronic Data Capture (REDCap) [25]. The purpose of the qualitative interviews was to allow Marshallese participants to describe dietary practices during pregnancy using their own words. The CBPR partnership developed the semi-structured interview guide through extensive fieldwork. A semi-structured interview guide with open-ended questions was used to encourage participants to speak openly while maintaining consistent inquiries across individual interviews. Broad questions were designed to encourage participants to speak openly, and probes were used to clarify nuances. The data collection activities were conducted simultaneously.

### 2.3. Data Collection

From March 2019 to March 2020, a purposive sample of 33 participants took part in the mixed methods study. Participants completed a survey and an individual interview. Data were collected in the participant’s language of choice, English or Marshallese. Three bilingual CHWs, trained in research methods and dietary information collection, facilitated individual interviews and survey data collection. Individual interviews were approximately 30 to 60 min in duration and were conducted either at The Center for Non-Profits Shop in Springdale, Arkansas or in the homes of the participants.

### 2.4. Data Analysis

Quantitative data analysis focused on descriptive statistics, including frequencies, percentages, means, and standard deviations. Descriptive statistics were computed for participant demographics, diet, and food insecurity.

Qualitative interviews were audio recorded and transcribed verbatim by a bilingual co-investigator. Transcripts were then translated from Marshallese to English and checked for accuracy by two female bilingual research staff. Qualitative data were analyzed using elements of Grounded Theory [22]. The CBPR team used Grounded Theory as a systematic methodology that involves inductive reasoning with first initial and then focused codes that lead to emergent themes [22]. All themes were collaboratively discussed in order to ensure scientific rigor and intercoder agreement and to develop the most salient themes within the data. There were two primary coders and one confirmation coder. Codes were classified in a codebook. Only the most representative quotes are being presented.

## 3. Quantitative Results

Table 1 shows participants’ demographic characteristics. Participants’ mean age was 28.1. A majority of the participants were married or in an unmarried partnership (84.9%). Twenty-four of the participants had a high school education or lower (72.7%), and 78.7% were unemployed and/or a student. A majority of the participants had no health insurance (60.6%), were born in the Marshall Islands (84.8%), and were not enrolled in The Special Supplemental Nutrition Program for Women, Infants, and Children (WIC) (63.6%). Participants’ mean number of pregnancies was 3.8, and 27.3% had experienced one or more miscarriages.

Table 2 shows participants’ dietary preferences. The majority of participants felt they had control over their gestational weight gain, exercise, and rest (97%). More than half of the participants felt they had control over their eating when watching television (54.5%); when there are different foods available (69.7); when at a party (57.6%); when high-fat foods are available (65.6%); when it is impolite to refuse (48.5); when pressured (63.6%); and when stressed (69.7%). A majority of the participants attend church more than two times a week (69.7%), and almost half of the participants said there were never healthy eating messages at church (42.4%). Less than a third of participants said they often had fruit for breakfast (18.8%) and had a vegetable at lunch (21.2%). Almost half of the participants stated that they consume one or more sodas a day (46.9%), and 75% said they had one or more sugary drinks per day.

Table 3 shows participants’ food insecurity. A majority of the participants said that paying for necessities such as food, housing, medical care, and electricity was somewhat to very hard (81.8%). When participants were asked if they had enough food to eat in their household, 15.2% said they often did not have enough to eat. Almost half of the participants said they or a member of their household received emergency food from a church, a food pantry, or a food bank or ate in a soup kitchen (48.5%).

## 4. Qualitative Results

Two themes emerged: (1) traditional beliefs about a healthy diet during pregnancy; and (2) dietary change during pregnancy. Participants are labeled with their participant identification numbers (PID).

### 4.1. Traditional Beliefs about a Healthy Diet during Pregnancy

Participants described traditional beliefs about a healthy diet and customs during pregnancy as encouraged by the elders in their community. Within this theme, four subthemes emerged: (1) should eat; (2) should not eat; (3) challenges to traditional diet; and (4) spiritual dietary customs during pregnancy.

#### 4.1.1. Should Eat

Participants described traditional Marshallese customs on what they should eat while pregnant. Participants said, “They (traditional elders) advise us to eat fruits and vegetables,” (PID 1); another said they were encouraged to “eat fruits and drink lots of water.” (PID 3). Participants also described being encouraged to eat more traditional foods from the islands, such as “Things like fish, pandanus, and drink coconut juice.” (10). Another participant was encouraged to eat “pandanus, coconut juice, and breadfruit. It’s also good because it’s part of our culture.” (PID 27). A majority of the traditional customary beliefs around what to eat were centralized on the health of the infant. For example, one participant said it is important to be “Really careful what you eat for the health of the baby.” (PID 4).

#### 4.1.2. Should Not Eat

In addition to traditional beliefs about what to eat, participants also described foods that their elders said they should not eat. A majority of the participants said that their elders told them not to eat salty, greasy, and fatty foods. For example, participants said they had been instructed not to eat “Fatty and salty foods,” (PID 45); “Salty and greasy foods.” (PID 3); “The fatty foods,” (PID 1); “I don’t need to eat lot of salty and fat food,” (PID 4); “They usually said not to eat food that are very salty,” (PID 5); “They used to say not to eat salty and fatty foods,” (PID 8); and “They say not to eat fried foods.” (PID 9).

Participants also discussed being told from their elders not to eat spicy foods. One participant said, “They tell me not to eat spicy foods,” (PID 12) and another said, “Foods that are spicy, like Hot Cheetos.” (PID 2). Refraining from spicy food and other unhealthy foods was to protect the infant. One participant said:

“My mom told me not to eat Hot Cheetos because it causes rashes. It can cause rashes for the baby. And I don’t drink a lot of soda. She tells me not to eat a lot of sweets because it will cause something to the baby. Sometimes I don’t understand.”

(PID 39)

Participants described being dissuaded from eating citrus or uncooked fish. One participant said:

“And they say not to eat limes and things like that. As for my mom, when she was pregnant with me she had too much lime and I got sick from it. My whole body was stiff and stomach as well, so those are the ones that they don’t want me to eat.”

(PID 12)

Although the Marshallese diet is traditionally fish-focused, participants described being encouraged to avoid fish, raw and cooked. One participant said that the elders encouraged her to avoid raw “Fish and seafood,” (PID 7), while another said to only eat “Cooked fish, not raw, because you get sick or make us sick.” (PID 5).

#### 4.1.3. Challenges to Traditional Diet

Despite the discussion of elders encouraging more traditional Marshallese diet choices during their pregnancies, participants discussed a lack of access to foods from their traditional diet. For example, one participant said, “In the islands I usually want pandanus, coconut juice, and breadfruit. It’s also good because it’s part of our culture. It is part of what we learn at our prenatal and hospital visits in our hospitals back home.” (PID 27). Another participant agreed and stated that she preferred “the local foods but we don’t have them here. Like bob pandanus and the type of banana called jilubuki.” (PID 15).

Challenges in traditional diet choices were also attributed to monetary limitations. One participant said, “We’re not able to buy them (referring to traditional food) because there is no money.” (PID 10). When participants were asked how difficult paying for necessities such as food was, 81.8% said it was somewhat to very hard. Additionally, when asked to describe the amount of food their household has to eat, 45.5% said they sometimes or often did not have enough to eat, and 48.5% said that they had used food assistance in the past year (Table 3).

#### 4.1.4. Spiritual Dietary Customs during Pregnancy

Participants discussed spiritual dietary customs surrounding pregnancy. One participant stated:

“In our customs we cannot eat at other places or eat other people’s food, especially here. They may do magic on us without knowing who did it. Other people usually warn other pregnant women and say things like ‘don’t go eating at other people’s houses because you don’t know what they will do to you.’”

(PID 12)

Another participant described a traditional custom when she said, “It’s inappropriate to walk and eat. Just sit and finish your food first. They say the baby’s cord will be tangled.” (PID 23). These traditional customs extended to beliefs on what might happen to the infant if the mother ingests certain foods. For example, one participant said that the elders told her to not “Eat raw fish. I don’t know maybe it’ll (the baby) bite us or something?” (PID 14). Another participant said that she was told not to eat octopus and this was because “They’re like kings, like demons of some sort. The demons will make us sick. I don’t know, that’s just what our elders would usually tell us.” (PID 15).

### 4.2. Dietary Change during Pregnancy

Within the dietary change during pregnancy theme, three subthemes emerged: (1) a healthy diet for my baby; (2) autonomy and diet; and (3) sugar-sweetened beverages.

#### 4.2.1. A Healthy Diet for My Baby

A majority of participants stated that they altered their diet to a focus on healthier options once they became pregnant, and this was for the health of their infant. One participant said, “Because the foods one eats goes directly to the baby.” (PID 2). Another participant stated, “We have to be healthy so the baby can be healthy.” (PID 1). One participant stated:

“Before I didn’t care what I ate because I was working a lot and I usually never had time to fix food for myself. That is what all the weight gain was about. Then when I found out I had anemia and low iron, which is what caused me to change because I want my baby to have a lot of blood for oxygen for his blood.”

(PID 39)

Another participant said, “Since I’m trying to eat for two people and give nutrition to my baby. I try not to limit the amount that I’m eating and eat healthy because I know that the baby is feeding on what I eat.” (PID 13).

The description of the participants’ shift in diet during pregnancy was very homogenous. A majority of participants described a diet of “Fruits and green salad with meats.” (PID 2). Another participant said: “I eat the kind of foods that are healthy for pregnant women such as fruits and vegetables, and steak. I really love steak.” (PID 12). However, when asked more specifically how often they ate fruits and vegetables, 81.3% stated they sometimes to never had fruit for breakfast, 78.8% stated that they sometimes to never had a vegetable at lunch, and 71.9% stated that they sometimes to never ate two or more vegetables at dinner (Table 2). Participants also consistently described eating rice and processed meats during their pregnancy, such as “I like to eat rice with spam,” (PID 4) and “Tuna, corned beef, and hot dogs,” (PID 17).

Similar to the homogenous discussion of dietary choices while pregnant, the participants described similar items that they removed from their diet once becoming pregnant, such as salty or greasy food and junk food. For example, one participant said, “I quit eating the foods that has salt and grease in them.” (PID 3). Another participant said, “I’m not eating as much junk food now, I am eating more real food.” (PID 23).

#### 4.2.2. Autonomy and Diet

Participants conveyed varying levels of autonomy in their dietary choice and gestational weight gain. Ninety-seven percent of the participants said that how much weight they gain is entirely up to them and they feel in control (Table 2). The concept of autonomy in diet control was mirrored in the qualitative response. For example, participants said, “I eat what I feel like eating,” (PID 23), “I eat what I want,” (PID 10), “I just eat whatever,” (PID 8), and “Anything you crave.” (PID 7). However, when asked about diet control more specifically, only about half of the participants felt in control. For example, a little more than half felt that they could control their diet when watching television (54.5%), when many different kinds of food are available (69.7%), when at a party (57.6%), when pressured by others (63.6%), when high-fat foods are available (65.6%), and when feeling stressed out or tired (69.7%) (Table 2).

#### 4.2.3. Sugar-Sweetened Beverages

About forty-seven percent of participants reported consuming at least one soda per day, and 75% of participants reported consuming at least one sugary drink per day during their pregnancy (Table 2). However, the participants’ qualitative responses demonstrated that many participants had reduced or eliminated Kool-Aid and soda from their diet. One participant said, “I stopped eating candies, Kool-Aid, and junk food. I also stopped drinking soda.” (PID 5). Another participant said, “I used to eat Kool-Aid and things like that when I had my morning sickness, but it affects my heart and I get chest pain from it.” (PID 23). Another participant agreed and stated, “I don’t eat Kool-Aid and the salty foods, or drink soda.” (PID 15). Much of the discussion surrounding removing Kool-Aid and soda from their diets was predicated on fear of harming their infant. For example, one participant said, “I often eat Kool-Aid and things like that but I haven’t since then. I don’t want anything to harm my baby. I stopped buying them. I completely stopped.” (PID 11).

## 5. Discussion

The findings from this study describe beliefs and practices during pregnancy among women who have migrated from the Marshall Islands to the US.

Participants discussed the traditional customs in Marshallese culture during pregnancy that are passed down from their elders. Much of this discourse was around the encouragement of eating fruits and vegetables and avoiding greasy and salty foods. Elders also encouraged eating traditional dishes. Most of the traditional recommendations are consistent with western nutritional guidelines. However, participants also described Marshallese customs that involve avoiding eating certain foods and eating behaviors based on spiritual folklore for the health of the infant. This finding is consistent with the previous literature that has identified pregnancy health folklore in Pacific Islander cultures that describe hexes and evil spells that can hurt the mother or infant [26,27,28,29].

While participants noted that their elders encouraged them to adhere to traditional practices, participants used individualistic discourse and described autonomy in their dietary practices. Almost all the participants (97%) stated that they had complete control over their weight, exhibiting an individualistic autonomy over their diet. These findings are similar to the recent literature with other Pacific Islander women who expressed a more individualistic conception of dietary practices, potentially demonstrating an acculturative shift [26,27,28,29]. However, the previous literature on this paradigmatic shift among Pacific Islanders has been identified within their native countries, and this study is the first study identifying a shift among women who have migrated to the US.

While participants discussed autonomy over their diet, they also discussed significant food insecurity; about 82% percent said that they struggled to pay for necessities such as food, and almost half reported that they sometimes or often did not have enough to eat and had used food assistance in the past year. This level of food insecurity among pregnant women is much higher than it has been documented in other populations [30,31,32]. The previous qualitative literature with the Marshallese in Arkansas has identified a strong desire for both healthy and traditional foods and financial barriers to obtaining them [19,33]. This is the first article to document this qualitative concern among pregnant Marshallese and the first study among pregnant Marshallese to document the high level of food insecurity risk using Hager et al.’s 2-item screening measure [34]. This finding demonstrates that it will be imperative to address the high level of food insecurity when designing dietary health educational interventions during pregnancy for Marshallese women.

Participants described the importance of having a healthy diet during pregnancy, and this was in order to have a healthy pregnancy, delivery, and infant. Both the traditional and individualistic perceptions of dietary practices were focused on the health of the infant. This finding is consistent with the previous literature demonstrating that Marshallese, and other Pacific Islander cultures, prioritize the infant throughout prenatal and postnatal decision making [6,26,35,36]. This understanding in Pacific Islander culture demonstrates the need to incorporate the health of the infant as a dominant behavior-guiding tool in educational and intervention programs for Marshallese women.

### Limitations and Strengths

This study’s findings should be evaluated with some limitations. All participants were recruited from Arkansas, and the results may or may not be generalizable to other Pacific Islander communities residing outside Arkansas. However, establishing evidence-based interventions designed for Pacific Islanders may also inform work with other indigenous populations who have strong collectivist cultures, thus increasing the generalizability of the proposed research [37,38,39,40,41]. The qualitative methods allowed participants to explore the research topic in their own words. The use of a CBPR approach is also a strength for these topics and population and is an asset to the study. However, in knowing the focus of the research, participants may have tempered their responses to make them more socially acceptable. Despite these limitations, this is the first study to document dietary practices during pregnancy among the Marshallese population residing in the US, thus adding substantially to the literature and filling a gap in knowledge.

## 6. Conclusions

To inform future interventions and better enable providers to care, this study aimed to understand dietary practices during pregnancy for Marshallese women who have migrated to the US. The continuation of some traditional customs is an indication of the value placed on cultural practices and the need for culturally-sensitive approaches in understanding post-migration behavioral changes on dietary practices while pregnant. However, the transition in discourse from traditional customs of dietary practices and spiritual folklore to an individualistic discourse highlights that acculturation is a complex and multifactorial process that should be included in maternal health education and interventions. The high level of food insecurity demonstrates the need to ensure that structural barriers to a healthy diet are addressed during behavioral interventions. The findings from this study are currently being incorporated into a maternal health intervention for Marshallese women to improve maternal and child health outcomes.

## Figures and Tables

**Table 1:** Participant demographics (N = 33).

	N	Percent of Sample (%)
**Age**	28.1	
**Marital Status**		
Single	5	15.2
Married	15	45.5
Divorced/separated	0	0
Widowed	0	0
A member of an unmarried couple	13	39.4
**Education**		
Never attended school or only attended kindergarten	0	0
Grades 1 through 8 (elementary)	4	12.1
Grades 9 through 11 (some high school)	9	27.3
Grade 12 or GED (high school graduate)	11	33.3
College 1 year to 3 years (some college or technical school)	9	27.3
College 4 years or more (college graduate)	0	0
**Household Size**	7.2	
**Employment**		
Employed for wages	7	21.2
Self-employed	0	0
Out of work for 1 year or more	10	30.3
Out of work for less than 1 year	11	33.3
Taking care of your family and home	4	12.1
Student	1	3
Retired/unable to work	0	0
**Health Insurance Status**		
No	20	60.6
Yes	13	39.4
**Birthplace**		
United States	5	15.2
Marshall Islands	28	84.8
Other	0	0
**WIC Status**		
No	21	63.6
Yes	12	36.4
**Number of Total Pregnancies**	3.8	
**Number of Miscarriages**	0.4	
0 miscarriages	24	72.7
1 miscarriage	6	18.2
2 miscarriages	3	9.1

Note: GED = graduate equivalency diploma; WIC = The Special Supplemental Nutrition Program for Women, Infants, and Children.

**Table 2:** Diet.

		N	Percent of Sample (%)
**How much weight I gain is entirely up to me**			
	Yes/Agree	32	97
	Maybe/Not sure	1	3
	No/Disagree	0	0
**If I eat properly and get enough exercise and rest, I can control my weight in the way I desire**			
	Yes/Agree	32	97
	Maybe/Not sure	1	3
	No/Disagree	0	0
**I can control eating when I am watching TV**			
	Yes/Completely sure	18	54.5
	Maybe/Not sure	2	6.1
	No/Not sure at all	13	39.4
**I can control eating when there are many different kinds of foods available**			
	Yes/Completely sure	23	69.7
	Maybe/Not sure	2	6.1
	No/Not sure at all	8	24.2
**I can control eating even when I am at a party**			
	Yes/Completely sure	19	57.6
	Maybe/Not sure	7	21.2
	No/Not sure at all	7	21.2
**I can resist eating even when high-fat foods are available (*n* = 32)**			
	Yes/Completely sure	21	65.6
	Maybe/Not sure	6	18.8
	No/Not sure at all	5	15.6
**I can resist eating even when I feel it’s impolite to refuse a second helping**			
	Yes/Completely sure	16	48.5
	Maybe/Not sure	8	24.2
	No/Not sure at all	9	27.3
**I can resist eating even when others are pressuring me to eat**			
	Yes/Completely sure	21	63.6
	Maybe/Not sure	5	15.2
	No/Not sure at all	7	21.2
**I can control eating when I feel stressed out or tired**			
	Yes/Completely sure	23	69.7
	Maybe/Not sure	2	6.1
	No/Not sure at all	8	24.2
**How often do you attend church or other religious meetings?**			
	Never	0	0
	Less than once a month	4	12.1
	1–2 times a month	5	15.2
	More than 2 times a month	23	69.7
	Don’t know/Not sure	1	3
**In an average month, how often does your church include any message encouraging healthy eating?**			
	Never	14	42.4
	Less than once a month	4	12.1
	1–2 times a month	4	12.1
	More than 2 times a month	10	30.3
	Don’t know/Not sure	1	3
**During the past three months, how often did you … eat fruit for breakfast? (*n* = 32)**			
	Often	6	18.8
	Sometimes	22	68.8
	Never	4	12.5
**During the past three months, how often did you … eat a vegetable at lunch?**			
	Often	7	21.2
	Sometimes	21	63.6
	Never	5	15.2
**During the past three months, how often did you … eat two or more vegetables at dinner? (*n* = 32)**			
	Often	9	28.1
	Sometimes	18	56.3
	Never	5	15.6
**Sodas consumed per day**			
	0	3	9.4
	More than 0 but less than 1	15	46.9
	1 or more	15	46.9
**Sugary drinks consumed per day (*n* = 32)**			
	0	2	6.3
	More than 0 but less than 1	6	18.8
	1 or more	24	75
**Height (in inches) (*n* = 32)**		60.0	
**Weight (in pounds) (*n* = 32)**		149.8	

**Table 3:** Food insecurity.

		N	Percent of Sample (%)
**How hard is it for you to pay for necessities like food, housing, medical care, and electricity?**			
	Very hard	10	30.3
	Somewhat hard	17	51.5
	Not hard at all	6	18.2
**Which of the following describes the amount of food your household has to eat?**			
	Enough to eat	18	54.5
	Sometimes not enough to eat	10	30.3
	Often not enough to eat	5	15.2
**In the last 12 months, did you or any member of your household ever get emergency food from a church, a food pantry, or a food bank, or eat in a soup kitchen?**			
	Yes	16	48.5
	No	16	48.5
	Don’t know/Not sure	1	3

## Data Availability

The deidentified data underlying the results presented in this study may be made available upon request from the corresponding author, Britni L. Ayers, at blayers@uams.edu. The data are not publicly available in accordance with funding requirements and participant privacy.

## Supplementary Materials

The following supporting information can be downloaded at: https://www.mdpi.com/article/10.3390/ijerph19116360/s1.
