# Supplementary material for: Dietary Practices during Pregnancy in a Marshallese Community: A Mixed Methods Analysis
Source: Int J Environ Res Public Health. 2022 May 24;19(11):6360. doi: 10.3390/ijerph19116360 (PMC9180099; doi:10.3390/ijerph19116360)
Supplement: Supplementary file 1 [file ijerph-19-06360-s001.zip › ijerph-1685471-supplementary.pdf]

PID Label

Early Pregnancy Survey
Pregnant Participants (PP)

Data Collector: ______________________________ **Date:** ______/______/__________

Demographic Survey

1. **What is your age?**
   (Jete am iiō?)

   ___ ___
   (Write age in years)
   (Je drettan iiō)

☐ Don’t know/Not sure (Jab jela/Jab kanooj jela)
If you are not sure, what do you estimate your age to be ________?
(Elañe kwōjjab kanooj jela, emaroñ jete am iiō?)
☐ Refused (Jab kōnaan uak)

2. **Are you...?**
   (Kwe ke juōn eo…?)

☐ Single (Ej make ian)
☐ Married (Emōj an mare)
☐ Divorced/Separated (Emōj an jebel)
☐ Widowed (Emōj an jako/mej eo ritūrūn)
☐ A member of an unmarried couple (Belele ak ejañin mare)
☐ Don’t know/Not sure (Jab jela/Jab kanooj jela)
☐ Refused (Jab kōnaan uak)

3. **Including yourself, how many people live in your home?**
   Ilo am kobaik kwe make, jete armij rej jokwe mweo imōm?)

Number of children (Uwaan ajiri ro) _______

Number of adults (Uwaan ritto ro) _______
☐ Don’t know/Not sure (Jab jela/Jab kanooj jela)
☐ Refused (Jab kōnaan uak)

4. **What is the highest grade you have completed?**
   (Kilaaj jete eo iļōñtata kwar kajemloke?)

☐ Never attended school or only attended kindergarten (Jañin kar jikuul ak kinder wōt)
☐ Grades 1 through 8 (Elementary) (Kilaaj 1 ñan 8, Elementary)
☐ Grades 9 through 11 (Some high school) (Kilaaj 9 ñan 11, high school jidrik)
☐ Grade 12 or GED (High school graduate)(Kilaaj 12 ak GED, kadiwōjlok jen High School)
☐ College 1 year to 3 years (Some college or technical school) (College 1 lok ñan 3 iiō, jidrik college ak ebbōk kilaaj ilo technical jikuul)
☐ College 4 years or more (College graduate) (College 4 iiō ak lablok, kadiwōjlok jen college)
☐ Don't know/Not sure (Jab jela/Jab kanooj jela)
☐ Refused (Jab kōnaan uak)

5. **Are you currently...?**
   Ilo tore in, kwōj ke…?)

☐ Employed for wages (Jerbal im bōk onāān)
☐ Self-employed (Jerbal ñan kwe make)
☐ Out of work for 1 year or more (Jañin jerbal iumin 1 iiō ak lablok)
☐ Out of work for less than 1 year (Jañin jerbal 1 iiō im driklok)
☐ Taking care of your family and home (Eo ej lale ak kōkajiriri ilo kabijuknen)
☐ A student (Juōn rijikuul)
☐ Retired (Juōn eo emōj an retire)
☐ Unable to work (Jab maroñ jerbal)
☐ Don't know/Not sure (Jab jela/Jab kanooj jela)
☐ Refused (Jab kōnaan uak)

6. **Do you have any kind of health care coverage, including health insurance, prepaid plans such as HMOs, or government plans such as Medicare? (Ewōr ke am jabdewōt kain joortoklik, ekobainjuren in takto, ko emoj kollaiki einwōt HMO ko, im injuren ko jen kien einwōt Medicare?)**

☐ Yes (Aet)
☐ No (Jaab)
☐ Don't know/Not sure (Jab jelā/Jab kanooj jelā)
☐ Refused (Jab kōnaan uak)

7. **Birthplace? (Jikin lotak)**
a. US Born (Amedka)
b. Marshall Islands (Majōl)
c. Other (Ijoko jet)
d. Don't know/Not sure (Jab jelā/Jab kanooj jelā)
e. Refused (Jab kōnaan uak)

8. **How long in US total? (Aetokan am bed Amedka)** ________________________
9. **Are you Marshallese? (Kwe ke ri Majōl?)**

☐ Yes (Aet)
☐ No (Jaab)

10. Are you enrolled in WIC? (Kwōj ke bed ilo burokram in WIC eo?)

☐ Yes (Aet)
☐ No (Jaab)
☐ Don't know/Not sure ((Jab jelā/Jab kanooj jelā)
☐ Refused (Jab kōnaan uak)

11. To your knowledge, are you now pregnant?
(Ilo am jela, kwōj bōroro ke kiio?)

☐ Yes (Aet)
☐ No (Jaab)

12. How many weeks pregnant are you? (Jete am wiik kiio?) ________
- ☐ Don't know/Not sure (Jab jelā/Jab kanooj jelā)
- ☐ Refused (Jab kōnaan uak)

13. Number of prenatal visits (Jete aḷen am taktō in bōroro) ________
- ☐ Don't know/Not sure (Jab jelā/Jab kanooj jelā)
- ☐ Refused (Jab kōnaan uak)

14. Number of pregnancies (Jete kattōn am bōroro?) ________
- ☐ Don't know/Not sure (Jab jelā/Jab kanooj jelā)
- ☐ Refused (Jab kōnaan uak)

15. Number of miscarriages (Jete kattōn an wōtlọk lọjeem?) ________________________
- ☐ Don't know/Not sure (Jab jelā/Jab kanooj jelā)
- ☐ Refused (Jab kōnaan uak)

Please describe your weight status during the following time period:
(Joij im kalikkar joñan eddoim ilo laajrak in tore kein:)

16. At present (Raan kein)

a. Underweight (Bed ilo joñan boun eo emera ak le lalin emman)
b. Normal (Bed ilo joñan boun eo emman)
c. Overweight (Bed ilo joñan boun eo eddo)
d. Very obese (Bed ilo joñan boun eo elap an eddo)
e. Don't know/Not sure (Jab jelā/Jab kanooj jelā)
f. Refused (Jab kōnaan uak)

17. How many pounds do you think is healthy for a woman to gain during pregnancy?
(Jete boun kwōj lomnak emman im ejmour nan an juōn kōra tobareeilo an bōroro?)

a. 10-20 pounds (10-20 boun)
b. 21-30 pounds (21-30 boun)
c. 31-40 pounds (31-40 boun)
d. 41-50 pounds (41-50 boun)
e. 51-60 pounds (51-60 boun)
f. Don't know/Not sure (Jab jelā/Jab kanooj jelā)
g. Refused (Jab kōnaan uak)

18. Did your doctor talk to you about weight gain?
(Takto eo am ear ke kōnaan ibbam kin eddolak in boun?)

a. Yes (Aet)
b. No (Jaab)
c. Somewhat (Jidik)
d. Don't know/Not sure (Jab jelā/Jab kanooj jelā)
e. Refused (Jab kōnaan uak)

If yes, how much weight did your doctor recommend that you gain? ________ lbs.
(Elañe aet, jete boun eo ear kalikkar ñan eok? __________boun

19. How many pounds do you expect YOU will gain during your pregnancy?
(Jete boun KWŌJ lōmnak kwōmaron eddoḷọk kake e ilo am bōroro?)

a. 10-20 pounds (10-20 boun)
b. 21-30 pounds (21-30 boun)
c. 31-40 pounds (31-40 boun)
d. 41-50 pounds (41-50 boun)
e. 51-60 pounds (51-60 boun)
f. Don't know/Not sure (Jab jelā/Jab kanooj jelā)
g. Refused (Jab kōnaan uak)

20. How many pounds do your friends and family tell you they think you should gain during pregnancy? (Jete boun eo ro jeram im nukum rej ba rej lemnak enaj emman ne kwoj tōbare ilo am bōroro?)

a. 10-20 pounds (10-20 boun)
b. 21-30 pounds (21-30 boun)
c. 31-40 pounds (31-40 boun)
d. 41-50 pounds (41-50 boun)
e. 51-60 pounds (51-60 boun)
f. Don't know/Not sure (Jab jelā/Jab kanooj jelā)
g. Refused (Jab kōnaan uak)

21. How would you feel if you gained 10-20 pounds during your pregnancy?
(Ewi am lōmnak ñe kwōnaj eddolọk 10-20 boun ilo tore eo kwōj bōroro?)

a. Extremely satisfied (Lukkun emman)
b. Neutral (neutral ak ejjelok oktak)
c. Extremely dissatisfied (Lukkun nana)
d. Don’t know/Not sure (Jab jelā/Jab kanooj jelā)
Refused (Jab kōnaan uak)

22. How would you feel if you gained 21-30 pounds during your pregnancy?
(Ewi am lōmnak ñe kwōnaj eddolọk 21-30 boun ilo tore eo kwōj bōroro?)

a. Extremely satisfied (Lukkun emman)
b. Neutral (Neutral ak ejjelok oktak)
c. Extremely dissatisfied (Lukkun nana)
d. Don’t know/Not sure (Jab jelā/Jab kanooj jelā)
e. Refused (Jab kōnaan uak)

23. How would you feel if you gained 31-40 pounds during your pregnancy?
(Ewi am lōmnak elañe kwōnaj eddolọk 31-40 boun ilo tore eo kwōj bōroro?)

a. Extremely satisfied (Lukkun emman)
b. Neutral (Neutral ak ejjelok oktak)
c. Extremely dissatisfied (Lukkun nana)
d. Don’t know/Not sure (Jab jelā/Jab kanooj jelā)
e. Refused (Jab kōnaan uak)

24. How would you feel if you gained 41-50 pounds during your pregnancy?
(Ewi am lōmnak elañe kwōnaj eddolọk 41-50 boun ilo tore eo kwōj bōroro?)

a. Extremely satisfied (Lukkun emman)
b. Neutral (Neutral ak ejjelok oktak)
c. Extremely dissatisfied (Lukkun nana)
d. Don’t know/Not sure (Jab jelā/Jab kanooj jelā)
e. Refused (Jab kōnaan uak)

25. How would you feel if you gained 51-60 pounds during your pregnancy?
(Ewi am lōmnak elañe kwōnaj eddoļọk 51-60 boun ilo tore eo kwōj bōroro?)

a. Extremely satisfied (Lukkun emman)
b. Neutral (Neutral ak ejjelok oktak)
c. Extremely dissatisfied (Lukkun nana)
d. Don't know/Not sure (Jab jelā/Jab kanooj jelā)
e. Refused (Jab kōnaan uak)

26. Please identify which infant you think looks the healthiest.
(Joij im kalikkar ewi ian pijain ajiri in im kwōj lōmnak ejmour tata.)

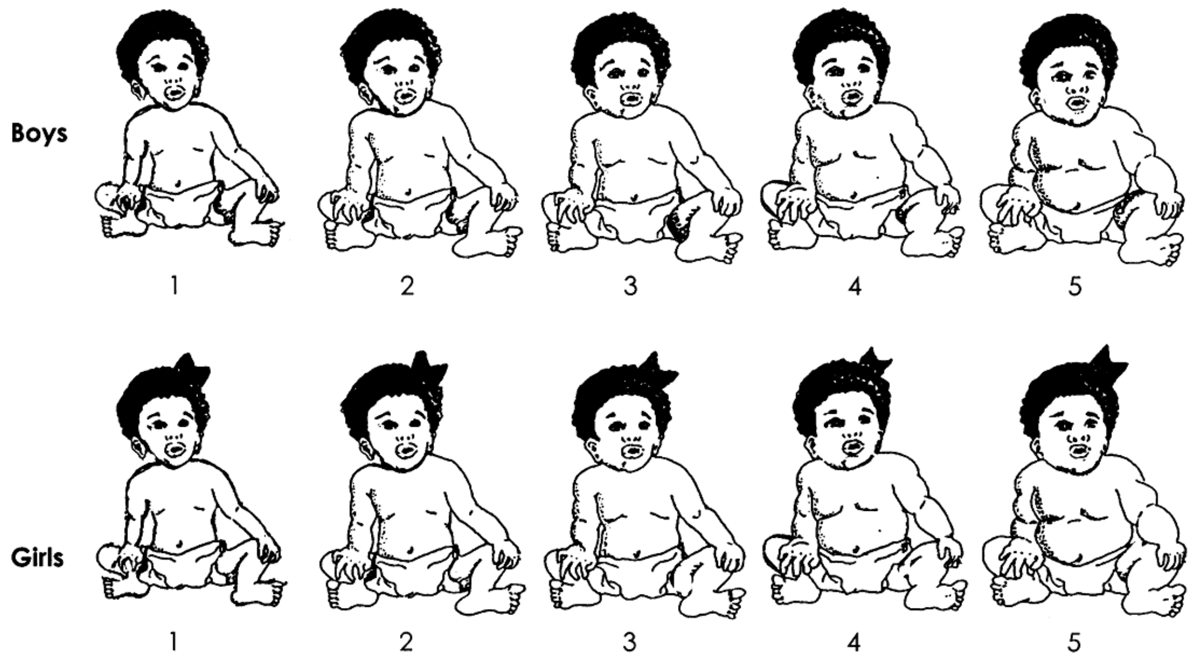

27. **Please identify which adult looks the healthiest.** **(Joij im kalikkar ewi wot ian ritto rein im kwōj lomnak ejmour tata.)**

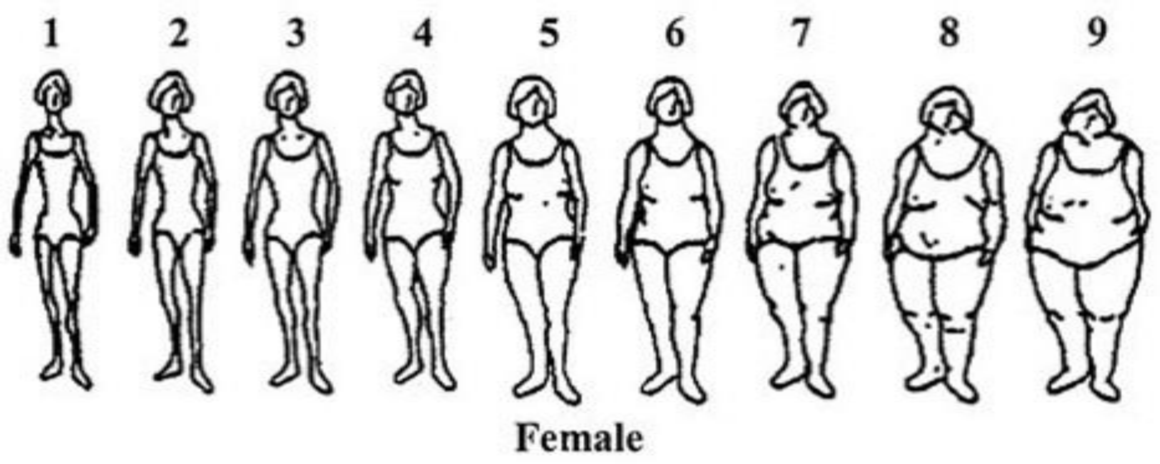

Eating and Exercise (Mona im Exercise)

Instructions: For each of the following statements, please choose **OFTEN**, **SOMETIMES**, or **NEVER** to answer and describe the amount of support your family and friends provide you with when starting and sticking to a healthy lifestyle, such as eating right and exercising.

(**Kōmelele ko:** Ilo kajjojo unin kenono kein, jouj im kelet **IKKUTKUT, JET IEN**, ak **JAÑIN** ñan uak kab kemeleleik ewi joñan an ro nukum kab ro mōttam rejañ eok ilo am jino kab rejañ eok bwe kwon dãbdeb wōt ilo am kōmman oktak ko ñan ejmour einwōt mōñā ko reōne kab exercise.)

	**Often (Ikkutkut)**	**Sometimes (Jet Ien)**	**Never (Jañin)**	**Don't know/Not sure (Jab jelā/Jab kanooj jelā)**	**Refused (Jab kōnaan uak)**
**28.** My family encourages me to start or stick with my exercise program. (Baamle eo ej rejañ io bwe in jinoe kab dābdeb ilo burokram in exercise ko.)	**Often (Ikkutkut)**	**Sometimes (Jet Ien)**	**Never (Jañin)**	**Don't know/Not sure (Jab jelā/Jab kanooj jelā)**	**Refused (Jab kōnaan uak)**
**29.** My family complains or makes fun of me for eating low-fat and low cholesterol foods. (Baamle eō ao ej kõmman keroro kab kajirere kōn ao mōñā mōñā ko edrik aer kūriij.)	**Often (Ikkutkut)**	**Sometimes (Jet Ien)**	**Never (Jañin)**	**Don't know/Not sure (Jab jelā/Jab kanooj jelā)**	**Refused (Jab kōnaan uak)**
**30**. My family reminds me not to eat high-fat foods. (Baamle eo aō ej kakemejmej iō bwe injab mōñā mōñā ko elab aer kuriij.)	**Often (Ikkutkut)**	**Sometimes (Jet Ien)**	**Never (Jañin)**	**Don't know/Not sure (Jab jelā/Jab kanooj jelā)**	**Refused (Jab kōnaan uak)**

**31.** My family also avoids eating the foods I am avoiding. (Baamle eo aō ejjab bar mōñā mōñā ko ijjab mōñā.)	**Often (Ikkutkut)**	**Sometimes (Jet Ien)**	**Never (Jañin)**	**Don't know/Not sure (Jab jelā/Jab kanooj jelā)**	**Refused (Jab kōnaan uak)**
**32.** My family criticizes or makes fun of me for exercising. (Baamle eo aō ej kwalok naan ko rejjab emman bareinwōt kajirere kōn aō exercise.)	**Often (Ikutkut)**	**Sometimes (Jet Ien)**	**Never (Jañin)**	**Don't know/Not sure (Jab jelā/Jab kanooj jelā)**	**Refused (Jab kōnaan uak)**

Instructions: The next two statements are about your attitude toward your own weight. For each statement, please choose one of the following: **YES/AGREE**, **MAYBE/NOT SURE**, or **NO/DISAGREE**.

Kōmlele ko: Melele kein ruo ej kin wawein am lōmnak kin eddoim make. Ñan kajjojo ian melele kein, joij im kalet juōn ian uak kein: **AET/ERRĂ**, **EMAROÑ/JAB LUKKUN JELĂ**, ak **JAAB/JAB ERRĂ**.

	**Yes/Agree (Aet/Errā)**	**Maybe/Not sure (Emaroñ/Jab Lukkun Jelā)**	**No/ Disagree (Jaab/Jab Errā)**	**Refused (Jab kōnaan uak)**
**33.** How much weight I gain is entirely up to me. (Ña eo ij eddoin joñan eddo eo ao.)	**Yes/Agree (Aet/Errā)**	**Maybe/Not sure (Emaroñ/Jab Lukkun Jelā)**	**No/ Disagree (Jaab/Jab Errā)**	**Refused (Jab kōnaan uak)**
**34.** If I eat properly and get enough exercise and rest, I can control my weight in the way I desire. (Elañe inaj kōjbarok ao mōñā, kabwe ien ao exercise im kakije, imaroñ kōjbarok joñan eddo eo ao.)	**Yes/Agree (Aet/Errā)**	**Maybe/Not sure (Emaroñ/Jab Lukkun Jelā)**	**No/ Disagree (Jaab/Jab Errā)**	**Refused (Jab kōnaan uak)**

Instructions: Now I am going to read to you some situations that may affect your eating habits. For each situation, please answer with **YES/COMPLETELY SURE**, **MAYBE/NOT SURE**, or **NO/NOT SURE AT ALL** to describe if these situations may affect your eating habits.

(**Kōmelele ko**: Kiō inaj itok bwe in riiti jet melele im remarōn jelōt wāwein am mōñā. Ñan kajojo melele kein, jouj im uaak **AET/ LUKKUN JELĀ, EMAROǸ/ JAB LUKKUN JELĀ**, ak **JAAB/ LUKKUN JAJE** ñan kalikkar ñe wāwein kein rej jelōt wāwein am mōñā.)

	Yes/Completely sure (Aet/Lukkun Jelā)	Maybe/Not sure (Emaroñ/Jab Lukkun Jelā)	No/Not sure at all (Jaab/ Lukkun Jaje)	Refused (Jab kōnaan uak)
**35.** I can control eating when I am watching T.V. (Imaroñ kōjbarok wāwein ao mōñā ilo ien ao alooj TV.)	**Yes/Completely sure (Aet/Lukkun Jelā)**	**Maybe/Not sure (Emaroñ/Jab Lukkun Jelā)**	**No/Not sure at all (Jaab/ Lukkun Jaje)**	**Refused (Jab kōnaan uak)**
**36.** I can control eating when there are many different kinds of foods available. (Imaroñ kōjbarok wāwein aō mōñā ilo ien an loñ kain mōñā.)	**Yes/Completely sure (Aet/Lukkun Jelā)**	**Maybe/Not sure (Emaroñ/Jab Lukkun Jelā)**	**No/Not sure at all (Jaab/ Lukkun Jaje)**	**Refused (Jab kōnaan uak)**
**37.** I can control eating even when I am at a party. (Imaroñ kōjbarok wāwein ao mōñā meñe ij bed ilo jikin bade ko.)	**Yes/Completely sure (Aet/Lukkun Jelā)**	**Maybe/Not sure (Emaroñ/ Jab Lukkun Jelā)**	**No/Not sure at all (Jaab/ Lukkun Jaje)**	**Refused (Jab kōnaan uak)**
**38.** I can resist eating even when high-fat foods are available. (Imaroñ rebij ao kōnaan mōñā meñe eloñ kain mōñā ko elab aer kuriij.)	**Yes/Completely sure (Aet/Lukkun Jelā)**	**Maybe/Not sure (Emaroñ/Jab Lukkun Jelā)**	**No/Not sure at all (Jaab/ Lukkun Jaje)**	**Refused (Jab kōnaan uak)**

**39.** I can resist eating even when I feel it's impolite to refuse a second helping. (Imaroñ rebij aō kōnaan mōñā meñe ijelā ke ejjab ekar ñan manit ilo ao jab bar mōñā.)	**Yes/Completely sure (Aet/Lukkun Jelā)**	**Maybe/Not sure (Emaroñ/Jab Lukkun Jelā)**	**No/Not sure at all (Jaab/ Lukkun Jaje)**	**Refused (Jab kōnaan uak)**
**40.** I can resist eating even when others are pressuring me to eat. (Imaroñ rebij aō kōnaan mōñā meñe ro jet rej akwelab bwe in mōñā.)	**Yes/Completely sure (Aet/Lukun Jelā)**	**Maybe/Not sure (Emaroñ/ Jab Lukun Jela)**	**No/Not sure at all (Jaab/ Lukun Jaje)**	**Refused (Jab kōnaan uak)**
**41.** I can control eating when I feel stressed out or tired. (Imaroñ kōjbarok wāwein ao mōñā ilo ien ien ko ij inebata ak mōk.)	**Yes/Completely sure (Aet/Lukun Jelā)**	**Maybe/Not sure (Emaroñ/Jab Lukun Jelā)**	**No/Not sure at all (Jaab/ Lukun Jaje)**	**Refused (Jab kōnaan uak)**

I would like to ask you some questions about your activities in the past month.

(Kio, ikōnaan kajjitōk jet kajjitōk ikijjien am makitkit im exerice ilo allōñ eo ej jemlok.)

Interviewer Note: Before asking, explain the difference between moderate and vigorous physical activity. An activity will only count in either moderate or vigorous, not both.

42. For the past month, about how often have you taken part in moderate physical activity (mowing your lawn, gardening, taking long walks, or playing sports) for at least 30 minutes?

(Ilo allōñ eo ej jemlok , ewi ikkutkut in am kōmani exercise ko rejjab kanooj ineddo einwōt [lawn mower, jerbal ilo atake ko, ak etetal aetok] tarrin 30 minit?)

☐ More than 4 times a week (Elōñlok jen 4 kōttan ilo juōn wiik)
☐ 2-4 times a week (2-4 kōttan ilo juōn wiik)
☐ About once a week (Juōn kōttan ilo juōn wiik)
☐ Rarely or never (Ejeja ak jañin)
☐ Don't know/Not sure (Jab jelā/Jab kanooj jelā)
☐ Refused (Jab kōnaan uak)

43. How does the amount of activity you have done since pregnant compare with your usual physical activity level?
Ewi oktakin jonan makitkit kane am kio jen ko kokujon kar kommani?

☐ More active (Laplok makitkit))
☐ Less active (Driklok makitkit))
☐ About the same (Joñan wōt juōn)
☐ Don't know/Not sure (Jab jelā/Jab kanooj jelā)
☐ Refused (Jab kōnaan uak)

44. How often do you attend church or other religious meetings?
(Ewi ikkutkut in am kobalak ilo ien jar ak kwelok ko an imōn jar ko?)

☐ Never (Jañin)
☐ Less than once a month (Iet lok jen 1 kōttan ilo juōñ allōñ)
☐ 1-2 times a month (1-2 kōttan ilo juōn allōñ)
☐ More than 2 times a month (Elōñ lok jen 2 kōttan ilo juōn allōñ)
☐ Don't know/Not sure (Jab jelā/Jab kanooj jelā)
☐ Refused (Jab kōnan uak)

45. In an average month, how often does your church include any message encouraging healthy eating?
(Iloan juōn allōñ, ewi ikkutkut in an kabun eo am kwalok naan in rōjañ ikkijen kilen kojbarok kijom?)

☐ Never (Jañin)
☐ Less than once a month (Iet lok jen 1 kōttan ilo juōn allōñ)
☐ 1-2 times a month (1-2 kōttan ilo juōn allōñ)
☐ More than 2 times a month (Elōñ lok jen 2 kōttan ilo juōn allōñ)
☐ Don't know/Not sure (Jab jelā/Jab kanooj jelā)
☐ Refused (Jab kōnaan uak)

During the past three months, how often did you…

(Iloan allōñ ko jilu remootlok, ewi ikkutkut in am kar…)

**46.** Eat fruit for breakfast? Mabun kin leen wojke?	**Often (Ikkutkut)**	**Sometimes (Jet ien)**	**Never (Jañin)**	**Don't know/Not sure (Jab jelā/Jab kanooj jelā)**	**Refused (Jab kōnaan uak)**

**47.** Eat a vegetable at lunch? (Mōña tarin juōn vegetable ilo ien mōñā in raelep?)	**Often (Ikkutkut)**	**Sometimes (Jet Ien)**	**Never (Jañin)**	**Don’t know/Not sure (Jab jelā/Jab kanooj jelā)**	**Refused (Jab kōnaan uak)**
**48.** Eat two or more vegetables at dinner? (Mōñā ruo ak lōñlok vegetable ilo ien mōñā in jota?)	**Often (Ikkutkut)**	**Sometimes (Jet Ien)**	**Never (Jañin)**	**Don’t know/Not sure (Jab jelā/Jab kanooj jelā)**	**Refused (Jab kōnaan uak)**

49. How hard is it for you to pay for necessities like food, housing, medical care, and electricity?
(Ewi joñan an ben ñan kwe ilo am kōllaik men ko raurok einwōt mōñā, imōn jokwe, taktō im jarom?)

☐ Very hard (Lukkun ben)
☐ Somewhat hard (Ebwe an ben)
☐ Not hard at all (Ejjab ben ñan jidrik)
☐ Don’t know/Not sure (Jab jela/Jab kanooj jela)
☐ Refused (Jab kōnaan uak)

50. Which of the following describes the amount of food your household has to eat?
(Ewi ian wawein kein ej kalikar joñan mōñā ilo kabijuknen eo am?)

☐ Enough to eat (Ebwe mōñā)
☐ Sometimes not enough to eat (Jet ien ej jabwe mōñā)
☐ Often not enough to eat (Ekka an jabwe mōñā)
☐ Don’t know/Not sure (Jab jela/Jab kanooj jela)
☐ Refused (Jab kōnaan uak)

51. In the last 12 months, did you or any member of your household ever get emergency food from a church, a food pantry, or a food bank, or eat in a soup kitchen?

(Iumin allōñ ko 12 remootlok, kwe ak ro ilo kabijuknen eo, kwar ke ebōk mōña ilo imōn jar ko, ak jikin ko ejjelok onaan mōñā ie einwōt (food pantry , food bank ak mōñā ilo soup kitchen ko?)

☐ Yes (Aet)
☐ No (Jaab)
☐ Don’t know/Not sure (Jab jela/jab kanooj jela)
☐ Refused (Jab kōnaan uak)

52. During the past 30 days, how often did you drink regular soda or pop that contains sugar? Do not include diet soda or diet pop.
(Iumin raan ko 30 remootlok, ewi ikkutkut in am idaak cola ak dren ko elōñ juka ie? Jab kobaik diet cola ilo uak ko am?)

____ times per day (jete kōttan ilo juōn raan)

____ times per week (jete kōttan ilo juōn wiik)

____ times per month (jete kōttan ilo juōn allōñ)

☐ Don't know/Not sure (Jab jela/jab kanooj jela)
☐ Refused (Jab kōnaan uak)

53. During the past 30 days, how often did you drink sugar-sweetened fruit drinks (such as Kool-Aid and lemonade), sweet tea, and sports drinks (such as Gatorade or Red Bull)? Do not include 100% fruit juice, diet drinks, or artificially sweetened drinks.
(Iumin raan ko 30 remootlok, ewi ikkutkut in am idaak dren ko emōj kōtōnal e (einwōt Kool-Aid kab lemonade, tea tōñal, kab dren ko nimen rikure (einwōt Gatorade ak Red Bull? Jab kobaik waj 100% fruit juice, diet cola, ak dren ko emōj kōtōñali kin juka in tōñal.)

____ times per day (jete kōttan ilo juōn raan)

____ times per week (jete kōttan ilo juōn wiik)

____ times per month (jete kōttan ilo juōn allōñ)

☐ Don't know/Not sure (Jab jela/jab kanooj jela)
☐ Refused (Jab kōnaan uak)
